# Identification of TPM2 and CNN1 as Novel Prognostic Markers in Functionally Characterized Human Colon Cancer-Associated Stromal Cells

**DOI:** 10.3390/cancers14082024

**Published:** 2022-04-16

**Authors:** Valentina Mele, Camilla Basso, Valeria Governa, Jesus F. Glaus Garzon, Manuele G. Muraro, Silvio Däster, Christian A. Nebiker, Robert Mechera, Martin Bolli, Alexander Schmidt, Roger Geiger, Giulio C. Spagnoli, Dimitri Christoforidis, Pietro E. Majno, Lubor Borsig, Giandomenica Iezzi

**Affiliations:** 1 Department of Biomedicine, University Hospital Basel and University of Basel, 4031 Basel, Switzerland; valentina.mele@unibas.ch (V.M.); manuele.muraro@usb.ch (M.G.M.); 2 Laboratory for Surgical Research, Ente Ospedaliero Cantonale, 6500 Bellinzona, Switzerland; camilla.basso@usi.ch; 3 Faculty of Biomedical Sciences, Università della Svizzera italiana, 6900 Lugano, Switzerland; dimitri.christoforidis@eoc.ch (D.C.); pietro.majno-hurst@eoc.ch (P.E.M.); 4 Department of Clinical Sciences Lund, Section of Oncology, Lund University, 221 85 Lund, Sweden; sweden.valeria.governa@med.lu.se; 5 Institute of Physiology, University of Zürich, 8006 Zürich, Switzerland; jesus.glausgarzon@hotmail.com (J.F.G.G.); lubor.borsig@uzh.ch (L.B.); 6 Department of General Surgery, University Hospital Basel, 4031 Basel, Switzerland; silvio.daester@usb.ch (S.D.); christian.nebiker@ksa.ch (C.A.N.); robert.mechera@gmail.com (R.M.); 7 Department of Visceral Surgery, Clarunis-University Center for Gastrointestinal and Liver Diseases, St. Claraspital and University Hospital Basel, 4002 Basel, Switzerland; martin.bolli@clarunis.ch; 8 Proteomics Core Facility, Biozentrum, University of Basel, 4056 Basel, Switzerland; alex.schmidt@unibas.ch; 9 Institute for Research in Biomedicine, Università della Svizzera italiana, 6500 Bellinzona, Switzerland; roger.geiger@irb.usi.ch; 10 Institute of Oncology Research, Università della Svizzera italiana, 6900 Lugano, Switzerland; 11 Institute of Translational Pharmacology, National Research Council, 00133 Rome, Italy; gcspagnoli@gmail.com; 12 Department of Surgery, Ente Ospedaliero Cantonale, 6500 Bellinzona, Switzerland

**Keywords:** colon cancer, tumor-associated stromal cells, proteomics, prognostic markers, TPM2, CNN1

## Abstract

**Simple Summary:**

Non-transformed cells of tumor microenvironment also impact on cancer outgrowth and progression. In colon cancer, a leading cause of cancer-related death worldwide, a high abundance of a heterogeneous cell population generally referred to as cancer-associated fibroblasts (CAFs) or tumor-associated stromal cells (TASCs) is associated with poor prognosis. The identification of TASC-specific markers could help to select patients for additional treatments and may provide novel targets for innovative therapies. Some markers have been proposed, but their prognostic significance is modest. We successfully expanded TASCs from human colon cancers and demonstrated their capacity to promote tumor growth and metastatic spread in vitro and in in vivo models. By comparing TASC whole protein expression, the so-called “proteome”, with that of stromal cells derived from matched healthy colon tissues, we identified two novel markers highly significantly associated with severe prognosis. Our results might help to identify patients at risk and might suggest new treatment options.

**Abstract:**

Stromal infiltration is associated with poor prognosis in human colon cancers. However, the high heterogeneity of human tumor-associated stromal cells (TASCs) hampers a clear identification of specific markers of prognostic relevance. To address these issues, we established short-term cultures of TASCs and matched healthy mucosa-associated stromal cells (MASCs) from human primary colon cancers and, upon characterization of their phenotypic and functional profiles in vitro and in vivo, we identified differentially expressed markers by proteomic analysis and evaluated their prognostic significance. TASCs were characterized by higher proliferation and differentiation potential, and enhanced expression of mesenchymal stem cell markers, as compared to MASCs. TASC triggered epithelial–mesenchymal transition (EMT) in tumor cells in vitro and promoted their metastatic spread in vivo, as assessed in an orthotopic mouse model. Proteomic analysis of matched TASCs and MASCs identified a panel of markers preferentially expressed in TASCs. The expression of genes encoding two of them, calponin 1 (CNN1) and tropomyosin beta chain isoform 2 (TPM2), was significantly associated with poor outcome in independent databases and outperformed the prognostic significance of currently proposed TASC markers. The newly identified markers may improve prognostication of primary colon cancers and identification of patients at risk.

## 1. Introduction

Tumor microenvironment (TME) includes a large number of nonmalignant cells decisively impacting cancer outgrowth and progression [1]. In particular, tumor stroma comprises heterogeneous cell populations of different origin, generally referred to as cancer-associated fibroblasts (CAFs), or tumor-associated stromal cells (TASCs) [2,3,4,5].

Colon cancer, a leading cause of cancer-related death worldwide [6], frequently presents a high stromal infiltration, which is associated with poor prognosis [7]. TASCs expanded from primary human colon cancers do enhance clonogenicity and migratory capacity of tumor cells in vitro through the release of soluble factors [8,9]. Furthermore, TASC-conditioned medium has been shown to decrease responsiveness of colon cancer cells to chemotherapeutic drugs in vitro [10], and co-injection of TASCs and tumor cells in vivo results in enhanced heterotopic tumor development [9,11].

Based on this background, the identification of stromal markers associated with colon cancer prognosis represents an active research field [12,13,14]. A variety of stroma-associated gene and protein signatures have indeed been reported to identify poor-prognosis colon cancer subtypes irrespective of tumor cell genetic profiles [13,15,16,17,18,19,20]. However, their clinical relevance is debated [21], possibly due to the high heterogeneity of phenotypic and functional profiles of colon-cancer-associated TASCs [8,9,11,14,22].

Proteomics technology has also been used to explore the expression of proteins associated with resistance to drug treatment in colon cancer cell lines and in sera from patients with colon cancer. In particular, markers emerging from murine models of colon cancer developing following administration of proinflammatory stimuli have been used for human studies [23]. However, these experimental approaches are typically modeling colitis-associated colon cancer [24], whereas, in sharp contrast, in humans, only a minority (<5%) of colon cancers are associated with inflammatory bowel diseases. Therefore, unsurprisingly, the described markers are of modest prognostic significance in human sporadic colon cancers, representing >85% of cases. Studies addressing proteome of TASCs expanded from human colon cancer surgical specimens have also been reported [16]. However, functional profiles of these cells were not provided and the prognostic relevance of the expression of emerging markers is unclear. 

To fill this knowledge gap, in this study, we expanded TASC populations from human sporadic colon cancers, representing a large majority of human tumors, and we performed a detailed analysis of their functional profiles in vitro. Most importantly, we evaluated their ability to promote tumor progression not only in conventional heterotopic models, but also in a newly established [25] orthotopic in vivo experimental model, allowing the analysis of their capacity to favor metastatic spread. Finally, these extensively characterized cells were used in proteomic studies, allowing the identification of novel markers of high prognostic relevance in human colon cancer.

## 2. Materials and Methods

### 2.1. TASC Isolation and Characterization

Clinical specimens of colon cancer tissues and corresponding autologous nontumor colon tissues were collected from consenting patients undergoing surgical treatment at the University Hospital Basel, St. Claraspital Basel, and Ospedale Civico Lugano, all in Switzerland, for sporadic colon cancer. The use of human samples was approved by local ethical authorities (Ethikkommission Nordwest und Zentralschweiz, EKNZ, study protocol n. 2014-388, and Comitato Etico Cantonale Ticino). 

Freshly excised specimens were mechanically minced and tissue fragments (approximately 2 × 2 mm) were plated in α-MEM (GIBCO) supplemented with 10% fetal bovine serum (FBS), 100 mM sodium pyruvate, 1% Kanamycin, 1% GlutaMAX-I (all from GIBCO), and FGF-2 (5 ng/mL, R & D Systems, Minneapolis, MN, USA). Outgrowing cells, usually detectable after one week of culture, were further expanded and characterized (see below).

To perform experiments reported in this study, we used eight different TASC preparations. From six of them, healthy mucosa-associated stromal cells, referred to as mucosa-associated stromal cells (MASCs), could also be expanded in sufficient numbers. Clinical-pathological characteristics of tumor samples from which TASCs (and MASCs) were derived are listed in Table S1.

TASC and MASC differentiation into adipogenic and osteogenic lineages, induced by using StemPro^®^ Adipogenesis Differentiation Kit and StemPro^®^ Osteogenesis Differentiation Kit (GIBCO), was evaluated upon Red Oil O and Alizarin Red staining (Sigma), as previously reported [26].

### 2.2. Tumor Cell Lines

Established human colon cancer cell lines (LS180, HCT116, and HT29) were purchased from European Collection of Cell Cultures (ECACC) and immediately stored in liquid nitrogen. Cells used for specific investigations were recovered from cryopreserved aliquots and cultured for a maximum of 10 passages. LS180 and HCT116 were maintained in RPMI-1640 supplemented with 10% FBS, GlutaMAX-I, nonessential amino acids (NEAA), 100 mM sodium pyruvate, 10 mM HEPES (all from GIBCO), and 50 mM 2-mercaptoethanol (Sigma-Aldrich, St. Louis, MO, USA), whereas HT29 was maintained in McCoy’s 5A medium (Sigma) supplemented with 10% FBS and GlutaMAX-I. 

Absence of mycoplasma contamination in cultured cells was verified by PCR testing prior to investigation. In specific experiments, LS180 cells co-expressing green fluorescent protein (GFP) and firefly luciferase (GFP/Luc-LS180 cells) [27] were also used.

### 2.3. Flow Cytometry and Cell Sorting

TASC phenotypes were analyzed upon staining with the antibodies listed in Table S2 by a dual laser BD FACS Calibur flow cytometer or a BD LSR Fortessa (BD Biosciences), following exclusion of dead cells based on propidium iodide incorporation, by using FlowJo software (Tree Star). 

Cells from cocultures were stained with EpCAM- or CD90-specific antibodies and analyzed by flow cytometry using a BD FACS Calibur. Absolute tumor cell numbers were assessed upon normalization of total cell counts to percentages of EpCAM + CD90- cells.

Alternatively, cells were sorted using a BD Influx cell sorter (BD Biosciences) for subsequent chemo-invasion and gene expression analysis. Dead cells were excluded based on DAPI incorporation. Purity of sorted cells was ≥ 99%.

### 2.4. Tumor–Stromal Cell Cocultures

Tumor cells were cultured alone or together with TASCs or MASCs at a 1:1 ratio in tumor cell medium for five days. In specific experiments, cultures were supplemented with anti-IL-6 (R&D Systems, 10 μg/mL) neutralizing antibodies. Following coculture, cells were stained with EpCAM- or CD90-specific antibodies and analyzed by flow cytometry or sorted for subsequent chemo-invasion assays using Matrigel-coated transwell plates, as previously described [27,28], or gene expression analysis. 

### 2.5. Gene Expression Analysis

Total cellular RNA was extracted and reverse transcribed from individual cell populations sorted by flow cytometry, as previously described [27]. Predeveloped Taqman^®^ assays (Applied Biosystems) were used to evaluate the expression of TWIST, SNAI1, SNAI2, ZEB1, E-cadherin (ECAD), and N-cadherin (NCAD) genes. The comparative CT method was used to quantify gene expression upon normalization using human glyceraldehyde-3-phosphate dehydrogenase (GAPDH) housekeeping gene as reference.

### 2.6. Hetero and Orthotopic Xenograft Models

In vivo experiments were approved by Basel Cantonal Veterinary office (license number 2266). NSG mice (NOD.Cg-Prkdcscid Il2rgtm1Wjl/SzJ) from Charles River Laboratories (Sulzfeld, Germany) were bred and maintained under specific pathogen-free conditions. 

In heterotopic tumor growth experiments, eight–tenweek-old NSG mice were injected subcutaneously (s.c.) in the flank with tumor cells alone, tumor cells admixed with equal numbers of TASCs, or tumor cells cultured with TASCs and sorted prior to injection (10^5^/mouse), resuspended in a 1:1 growth factor reduced Matrigel (BD Biosciences)/PBS solution. Tumor formation was monitored twice weekly by palpation and caliper measurements. After 4 weeks, all mice were sacrificed and tumors were harvested. Tumor volumes (in mm^3^) were determined according to the formula (length × width^2^)/2.

Formalin-fixed cryosections (10 μm) from each tumor were incubated with Cy3-conjugated mouse anti-alpha smooth muscle actin (αSMA) (Sigma-Aldrich) and rat anti-CD31 (BD Biosciences) antibodies, followed by secondary species-specific Alexa Fluor 488-conjugated antibodies (Invitrogen, Waltham, MA, USA) or mouse anti-human EpCAM (Cell Signaling), followed by secondary species-specific Alexa Fluor 546-conjugated antibodies (Invitrogen, Waltham, MA, USA). Fluorescence microscope images were captured with 10× and 20× magnification using a digital camera and analyzed by AnalySIS software (Soft Imaging System GmbH). Microvessel density (MVD) was evaluated as previously described [27].

In the orthotopic tumor model [25], eight–ten-week-old NSG mice were injected intra cecum (i.c.) with 105 GFP/Luc-LS180 cells alone or admixed with equal numbers of TASCs, or with LS180 cells previously cocultured with TASCs, and subsequently sorted. Similarly to heterotopic studies, cells were resuspended in a 1:1 growth factor reduced Matrigel (BD Biosciences)/PBS solution. Tumor development was monitored weekly after injection using the LB983 NightOWL II imaging system (Berthold Technologies GmbH) [28]. After 4 weeks, animals were sacrificed and tumors, blood, spleens, livers, and lungs were harvested. 

Metastatic foci in spleens, livers, and lungs were assessed macroscopically, by histological and immunofluorescence analysis, or, following tissue digestion, through detection of EpCAM/GFP-positive cells by flow cytometry. 

### 2.7. Quantitative Proteomics

TASCs or MASCs (10^6^/sample) were lysed in 2% sodium deoxycholate, 100 mM ammonium bicarbonate, reduced, alkylated, and digested with sequencing-grade modified trypsin (1/50, w/w; Promega, Madison, Wisconsin, WI, USA). After desalting and drying under vacuum, peptide samples were labelled with isobaric tag (TMT 10-plex, Thermo Fisher Scientific, Waltham, MA, USA), as described [29], desalted, dried under vacuum, and subsequently subjected to HPLC fractionation and LC-MS/MS analysis using a Q Exactive Orbitrap Analyzer coupled to an Easy-1000 LC system (both Thermo Fisher Scientific), and applying the same MS parameters as previously illustrated [29]. Data were searched using the MASCOT algorithm (Matrix Science, Version 2.4.1) against a decoy database containing normal and reverse sequences of the predicted SwissProt entries of Homo sapiens (www.ebi.ac.uk, release date 24 November 2014, total number of entries: 84610,accessed date: 9 March 2021). Results were imported into the Scaffold Q+ software (Proteome Software, Inc), and protein false discovery rate (FDR) was calculated by the scaffold local FDR algorithm and set to 1%, based on the number of decoy hits. Acquired reporter ion intensities in the experiments were employed for automated quantification and statistical analysis using a modified version of our in-house developed SafeQuant R script. All ratios were corrected for ratio distortion using the spiked-in protein calibration mix [29]. Raw mass spectrometry data used in this study and the Mascot analysis files are available via ProteomeXchange (accession code PXD005796, Username: reviewer25764@ebi.ac.uk, Password: vadeGPHV).

### 2.8. Statistical Analysis

Statistical analyses were performed using Fisher, Wilcoxon matched pairs, and Mann–Whitney tests, as appropriate, using GraphPad Prism 5 software (GraphPad Software) or SPSS version 22.0 software (IBM Corporation). *p* values ≤ 0.05 were considered significant.

Prognostic significance of the expression of defined genes was analyzed by using Xena (https://xenabrowser.net) website and GDC TCGA COAD database with primary tumor samples (accessed date: 14 January 2022).

## 3. Results

### 3.1. Isolation and Characterization of TASCs from Human Primary Colon Cancers

**Figure 1:**
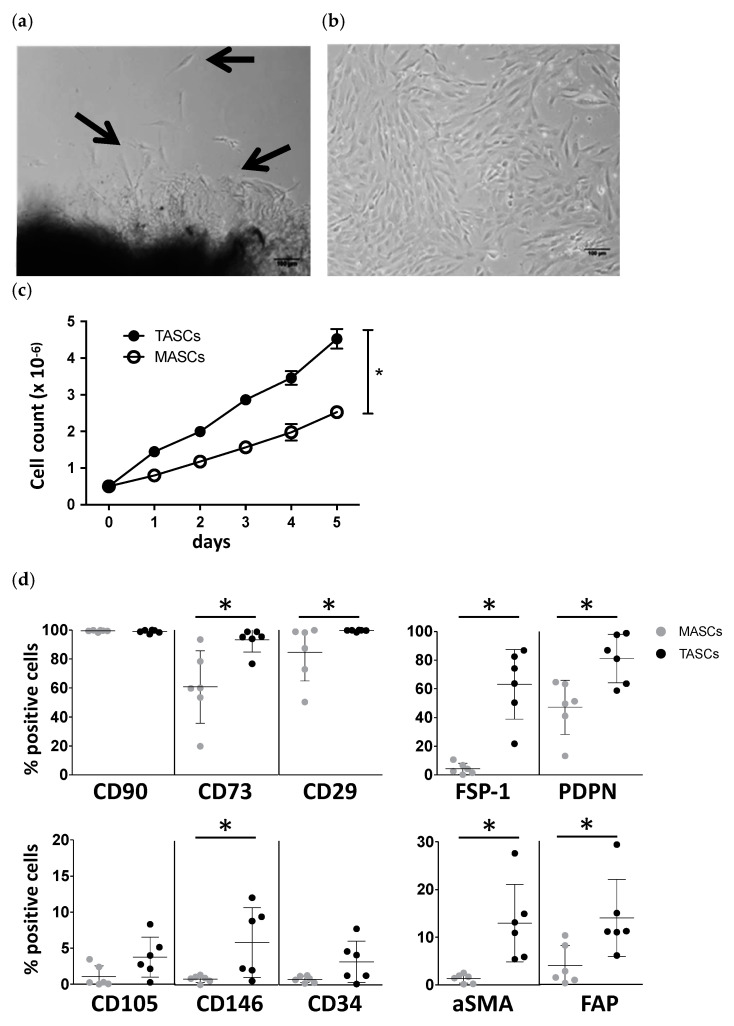
Isolation and characterization of TASCs from primary sporadic colon cancers. (**a**) Representative TASC outgrow from tumor tissues two days after isolation. Arrows indicate cells outgrowing from tissue fragments. (**b**) Morphological appearance of TACs after 2-week culture. Magnification 20×; scale bar 100 μm. (**c**) Growth kinetics of stromal cells isolated from tumor (TASCs) or from corresponding healthy colonic mucosa (MASCs). Data refer to three independent experiments performed with different TASC/MASC matched preparations. * *p* ≤ 0.05. (**d**) Phenotypic analysis by flow cytometry of TASCs and corresponding MASCs isolated from six different colon cancer samples. Percentages of cells positive for the indicated markers are reported Means ± SD are indicated by bars. Statistical significance of differences between TASCs and MASCs was evaluated by Wilcoxon signed rank test, * *p* ≤ 0.05.

Freshly excised specimens of human colon cancer samples and corresponding adjacent nontumoral colonic mucosa (*n* = 32) were processed for isolation of stromal cells, as detailed above. TASCs outgrew in 28 samples (87.5%) and could be expanded in vitro up to 12 passages (Figure 1a,b). In contrast, stromal-like cells from tumor-free mucosa tissues, hereafter referred to as mucosa-associated stromal cells (MASCs), could only be expanded from 14 samples (43.8%, Fisher test, *p* < 0.001) and displayed a significantly reduced proliferation rate as compared to TASCs (Figure 1c).

From six of those, healthy mucosa-associated stromal cells, referred to as mucosa-associated stromal cells (MASCs), could also be expanded in sufficient numbers. 

Representative stainings are shown in Figure S1a (see Supplementary Materials), whereas Figure 1d reports data from all samples under investigation. Phenotypic analysis showed that both TASC and MASC cell populations expressed CD90, a known stromal cell marker. However, additional markers, such as CD73 and CD29 [30], were expressed to significantly higher extents in TASC. TASCs, but not MASCs, also included small subpopulations positive for CD146, CD34, and CD105 markers. Putative CAF markers, including fibroblast-specific protein 1 (FSP-1), podoplanin (PDPN), a-smooth muscle actin (a-SMA), and fibroblast activation protein (FAP), were also expressed by significantly higher cell percentages of TASCs compared to MASCs, although to variable extents across different samples. No expression of the hematopoietic marker CD45 nor of the endothelial marker CD31 was ever observed.

The differentiation potential of TASCs and MASCs was also assessed. TASCs consistently displayed the capacity to differentiate into adipogenic and osteogenic lineages (Figure S1b), thus indicating that they do comprise multipotent cells. Instead, MASCs did not survive for the entire culture period required to achieve differentiation and only in one out of four tested samples could we observe a minor degree of adipo- and osteocyte differentiation.

Thus, cultured TASCs are characterized by a different phenotypic profile, and proliferation and differentiation potential, as compared to similarly cultured MASCs.

### 3.2. Functional Potential of Cultured TASCs 

**Figure 2:**
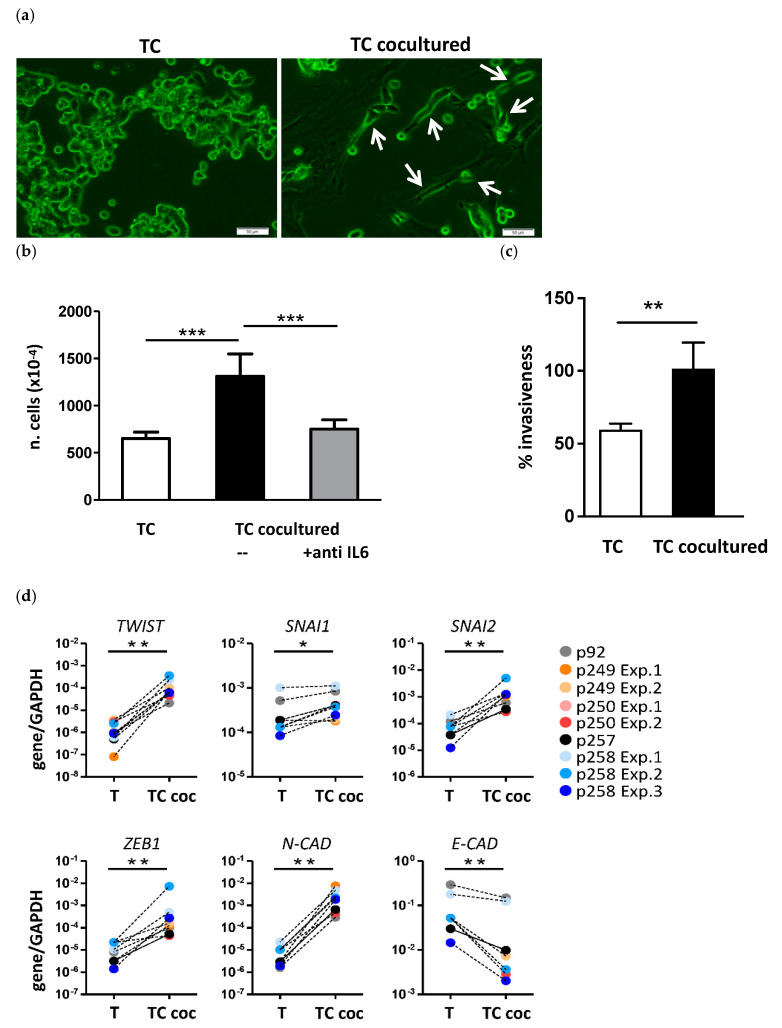
TASCs promote proliferation and induce EMT in colon cancer cells. (**a**) Morphological modifications observed in GFP/Luc-LS180 cells cultured alone (TC) or with TASCs (TC cocultured) and sorted upon EpCAM staining. Arrows highlight representative events. Magnification 20×; scale bar 50 μm. (**b**) Numbers of LS180 cells cultured alone (TC) or with TASCs (TC cocultured) in the presence or absence of 10 μg/mL anti-IL6 neutralizing antibody. Data refer to 8 independent experiments performed with two different TASC preparations (Mann–Whitney test, *** *p* ≤ 0.001). Cell numbers were normalized based on percentages of EpCAM+ and CD90+ cells. (**c**) Invasive capacity of LS180 cells cultured alone (TC) or with TASCs (TC cocultured), as assessed by chemoinvasion assay. (**d**) LS180 cells were cultured alone (TC) or together with TASC (TC coc) isolated from six different tumor samples. EpCAM+ cells were then sorted from cocultures and the expression of the indicated EMT-related genes was assessed by quantitative RT-PCR analysis. Statistical significance was evaluated by Mann–Whitney test (* *p* ≤ 0.05, ** *p* ≤ 0.01).

To explore functional features of cultured TASCs, we first evaluated their effects on colon cancer cells in vitro. Upon coculture with TASCs, colon cancer cells acquired an elongated shape (Figure 2a). Moreover, in agreement with previous reports [27], coculture with TASCs resulted in increased proliferation of EpCAM+ tumor cells, mediated by IL-6 (Figure 2b), and higher invasive capacity (Figure 2c). 

To further confirm the functional potential of cultured TASCs, we evaluated their ability to promote EMT in tumor cells, as previously demonstrated for bone-marrow-derived stromal cells [27]. Following culture in the presence of TASCs, EpCAM+ tumor cells were sorted and their expression of EMT-related genes was quantified by qRT-PCR. Expression of TWIST, SNAI1, SNAI2, and ZEB1 was significantly increased in cocultured cells, as compared to cultures performed in the absence of TASCs. Importantly, following coculture, upregulation of the mesenchymal marker N-cadherin, accompanied by a ≥4-fold downregulation of the epithelial marker E-cadherin, was also observed (Figure 2d). These phenomena were consistently induced by different TASC preparations (Figure 2d) in different colon cancer cell lines (Figure S2).

Interestingly, the capacity of TASCs to trigger EMT in colon cancer cells appeared to depend on their multipotent state, since upregulation of EMT-related genes in tumor cells was significantly reduced if TASCs were previously differentiated towards adipogenic or osteogenic lineages (Figure S3). EMT induction was undetectable upon culture of tumor cells in the presence of TASC-conditioned medium or in transwell plates (Figure S4), consistent with an indispensable cell-to-cell contact requirement, as previously reported for bone-marrow-derived stromal cells [27].

### 3.3. TASC-Mediated Effects on Tumor Growth and Metastasis Formation In Vivo

**Figure 3:**
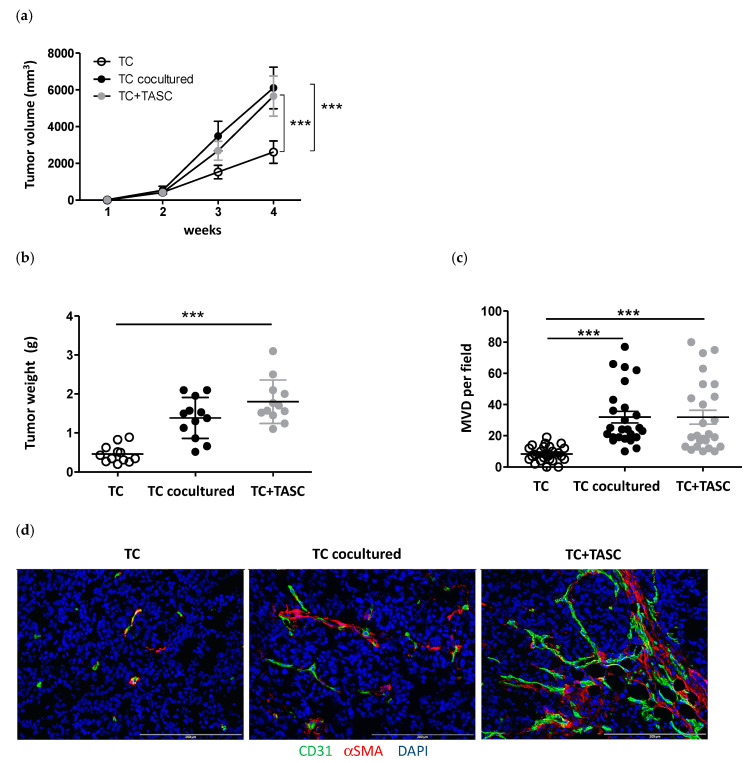
TASCs promote tumor development in a heterotopic tumor xenograft model. (**a**) Growth kinetics of tumors developed upon s.c. injection of tumor cells alone (TC), cocultured with TASCs and sorted prior to injection (TC cocultured), or cultured alone and admixed with TASCs prior to injection (TC+TASC). Mean values ± SD from three independent experiments performed with different TASCs (total mice injected *n* = 12/experimental group) are reported (Mann–Whitney test, *** *p* ≤ 0.001). (**b**) Tumor weights measured at the end of the experiment. Individual values from three independent experiments (mice injected *n* = 12/experimental group) are shown. Means ± SD are indicated by bars (Mann–Whitney test, *** *p* ≤ 0.001). (**c**) Microvessel density (MVD) quantification in developed tumors. Numbers of CD31+ cells per field are reported. Data refer to individual values from three independent experiments (*n* = 12 mice/experimental group, 2 fields/tumor). Means ± SD are indicated by bars (Mann–Whitney test, *** *p* ≤ 0.001). (**d**) Representative immunofluorescence staining of CD31 (green), α-SMA (red), and DAPI (blue) on tumor sections. Magnifications 20×, scale bar 100 μm.

To address the effects of cultured human TASCs on tumor cells in vivo, we first used a heterotopic tumor model based on subcutaneous (s.c.) tumor cell injection. Implantation of colon cancer cells either preconditioned with TASCs and then sorted prior to injection, or admixed with TASCs immediately before injection led to the formation of larger tumor masses, as compared to tumor cells cultured and injected alone (Figure 3a–c), which were also characterized by a higher vascularization, as detectable by CD31-specific staining (Figure 3d).

**Figure 4:**
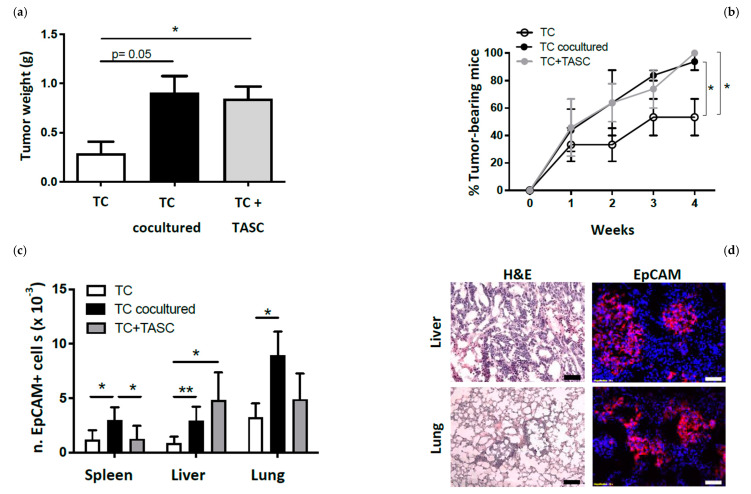
TASCs support growth and metastatic spread of orthotopic tumor xenografts. (**a**) Tumor incidence in NSG mice injected i.c. with GFP/Luc-LS180 alone (TC), cocultured with TASCs and sorted prior to injection (TC cocultured), or cultured alone and admixed with TASCs prior to injection (TC+TASC). Data (means ± SD) refer to two experiments performed with TASCs isolated from two different colon cancer samples (*n* = 9/experimental group). (**b**) Tumor weights as measured four weeks after injection. Data (means ± SD) refer to two experiments performed with TASCs isolated from two different colon cancer samples (*n* = 9/experimental group). Statistical significance was evaluated by Mann–Whitney test (* *p* ≤ 0.05). (**c**) Numbers of EpCAM+ cells detected by flow cytometry in spleens, livers, and lungs of NSG mice injected as detailed above (*n* = 9/group; Mann–Whitney test, * *p* ≤ 0.05, ** *p* ≤ 0.01). (**d**) Representative histological analysis by hematoxylin and eosin (H&E) and immunofluorescence staining of EpCAM (red) of metastatic foci in livers and lungs. Magnification 20×, scale bar 100 μm.

Notably, however, heterotopic in vivo models poorly mirror clinical colon cancer specificities, since they do not allow a reliable evaluation of metastatic tumor spread. To address more effectively these issues, we developed an orthotopic colon cancer model [25] where tumor cells, treated as detailed above, were inoculated in the cecal wall of NSG mice, and tumor formation was monitored over time based on luciferase activity (Figure S5a). Injection of colon cancer cells precultured with or admixed with TASCs resulted in higher tumor incidence and in the development of larger tumor masses (Figure 4a,b). Most importantly, upon orthotopic injection of tumor cells preconditioned by or admixed with TASCs, significantly higher numbers of colon cancer cell were shown to metastasize into distant organs, as indicated by detection of EpCAM+ cells by flow cytometry (Figure S5b and Figure 4c) and immunofluorescence (Figure 4d).

### 3.4. Identification of TASC-Specific Protein Markers

Taken together, our data from in vitro and in vivo assays provided a powerful validation of identity and functional role of cultured colon-cancer-derived TASCs. A variety of markers from colon cancer-associated stromal cells have previously been identified at the gene and protein expression level [13,15,16,17,18,19,20]. However, their prognostic significance is modest, and the identification of additional, clinically more relevant markers is urgently required. 

Therefore, we utilized HPLC fractionation and LC-MS/MS analysis to explore the proteome of colon cancer-derived cultured TASCs in comparison to that of matched MASCs.

**Figure 5:**
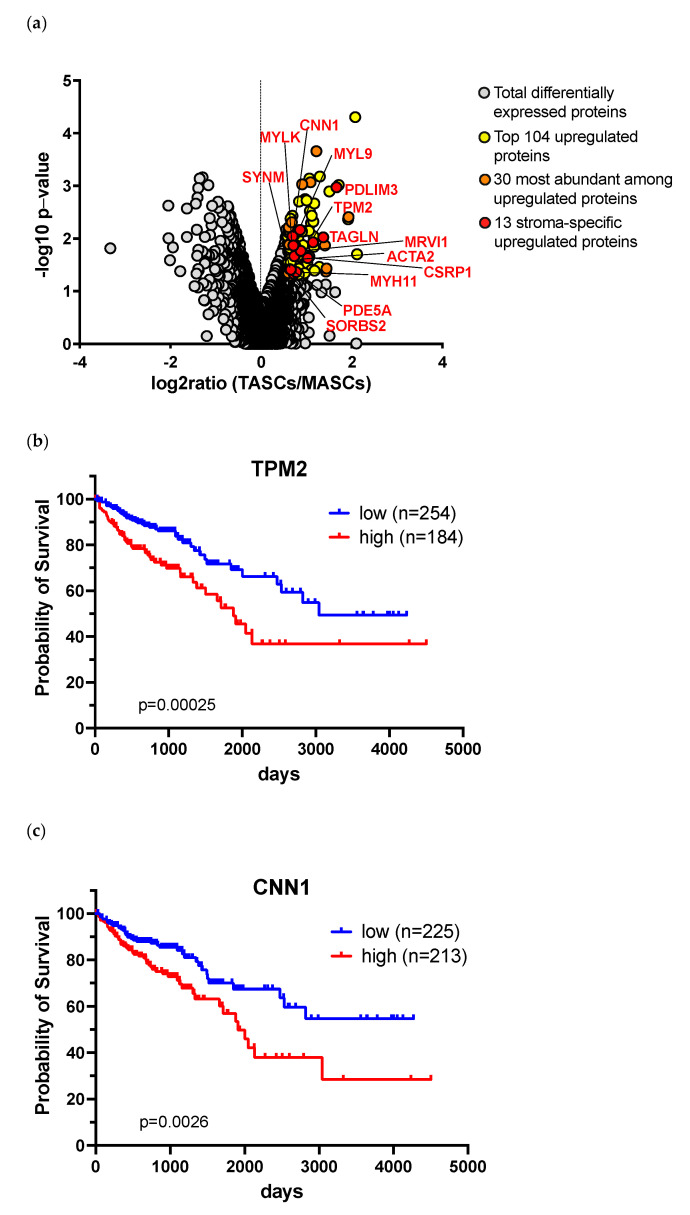
Proteomic analysis identifies proteins overexpressed in TASCs as compared to MASCs of high prognostic significance. (**a**) Volcano plot illustrating proteins overexpreseed in TASCs as compared to matched MASCs. (**b**,**c**) Kaplan–Meier plots showing survival probability in colon cancers with high (red line) or low (blue line) expression of (**b**) TPM2 or (**c**) CNN1 in colon cancer samples of the TCGA COAD cohort (*n* = 438), classified according to best expression cut-off. *p* values were estimated by log-rank test.

Among >6900 proteins identified in both cell types, 402 were differentially expressed (Figure 5a) and 104 of them were significantly upregulated in TASCs (fold increase >1.5, *p* value < 0.05, Table S3). 

Among the 30 most abundantly expressed proteins, 13 were represented by proteins predominantly expressed by cells of stromal lineages, according to publicly available databases (proteinatlas.com) (Table 1).

They included proteins involved in contractile functions, such as transgelin (TAGL), a-SMA, calponin 1 (CNN1), sorbin and SH3 domain-containing protein 2 (SORBS2), and PDZ and LIM domain protein 3 (PDLIM3); cytoskeletal proteins, such as synemin (SYNM); myosin proteins, including tropomyosin beta chain isoform 2 (TPM2), myosin regulatory light polypeptide 9 (MYL9), myosin 11, and myosin light chain kinase (MYLK); cysteine and glycine-rich protein 1 (CSRP1), and phosphodiesterase-5A (PDE5A), involved in cellular differentiation and regulation of intracellular concentrations of cyclic nucleotides, respectively.

TASCs also expressed additional previously reported stromal cell markers, including calmodulin 1 (CDN), transglutaminase 2 (TGM), periostin (POSTN), insulin-like growth factor binding protein 7 (IGFBP7), TFG-beta (TGFB1) and, microtubule-associated protein 1B (MAP1B). However, they were not expressed to significantly higher extents in TASCs than in MASCs (Table S4).

### 3.5. Comparative Analysis of the Prognostic Significance of TASC-Specific Markers

Some of the identified proteins, including TAGL and a-SMA, have been previously proposed as TASC markers predictive of clinical course of colon cancers (12, 14–19). Thus, the identification of additional proteins overexpressed in TASCs as compared to MASCs urged us to explore their prognostic significance. We, therefore, took advantage of public available databases to evaluate the expression of the corresponding genes in comparison with genes encoding previously reported TASC proteins. 

Analysis of the COAD TCGA cohort, including 438 human primary colon cancers with corresponding clinical data, revealed that expression above the median value of most previously reported TASC markers genes, including LOXL2, podoplanin (PDPN), FAP, FSP-1, CALD1, POSTN, IGFB7, BGN, and PLOD2, is devoid of prognostic significance. High a-SMA and TAGLN gene expression only showed a trend suggesting an association with severe prognosis (Table 1). In contrast, among newly identified TASC-specific markers, expression of TPM2, CNN1, and MYL9 genes is significantly associated with decreased overall survival (*p* = 0.0063, 0.0053, and 0.045, respectively, Table 1 and Figure 5b,c).

When best cut-off values were used for analysis, expression of a-SMA and TAGLN genes, as well as of SYNM, MRVI1, and CSRP1 genes, also showed a significant correlation with worse survival. However, expression of TPM2 and CNN1 still displayed the strongest significance, thus possibly indicating that these markers may represent superior prognostic markers.

## 4. Discussion

Accumulating evidence indicates that TASCs critically contribute to the aggressiveness of human colon cancer [7]. 

A number of TASC-associated markers have been proposed for molecular classification and prognostication of colon cancers [11,15,16,17,18,19,20]. However, their clinical relevance remains modest, possibly due to their original characterization in experimental models poorly mimicking human colon cancer or due to an insufficient functional evaluation of target stromal cells. Indeed, the combination of phenotypic and functional studies has recently been recommended as a critical prerequisite for the investigation of novel clinically significant TASC markers [4]. 

To address the identification of novel markers of potentially higher clinical relevance, we isolated TASCs from human primary colon cancer tissues and MASCs from matched tumor-free colonic tissues, and, upon in vitro expansion, we extensively characterized their phenotypic and functional profiles. Most importantly, functional studies included the use of a recently developed orthotopic colon cancer model, allowing the evaluation of both local growth and metastatic spread.

Expanded TASCs included high percentages of cells expressing markers previously reported as CAF-specific, such as FSP-1, podoplanin, α-SMA, and FAP [8,10,16,17], as compared to MASCs. Moreover, sizeable percentages of cells positive for the putative stemness markers CD146 and CD34 [30] were also detected among TASCs but not MASCs, and the differentiation potential of cells used in our study could be successfully demonstrated.

In keeping with previous reports [31,32], TASCs promoted colon cancer cell proliferation through IL-6 secretion. Furthermore, in their undifferentiated state, but not following predifferentiation towards adipogenic or osteogenic lineages, they were able to induce EMT in colon cancer cells, suggesting that EMT-induction ability might represent a peculiar property of multipotent progenitors comprised within TASCs.

More interestingly, our study demonstrates for the first time a direct impact of patient-derived TASCs on colon cancer development and metastasis formation in vivo in an orthotopic experimental model, possibly supported by increased tumor vessel density. 

Notably, in vitro pre-exposure of colon cancer cells to TASCs was sufficient to induce long-term modifications of tumor cell behavior in vivo, consistent with activation of intrinsic biological processes in cancer cells, such as during EMT. In previous studies, we have documented similar properties in bone-marrow-derived mesenchymal stromal cells, thereby further underlining similarities between TASCs and cells endowed with stem-cell-like features.

A slight difference was observed in the metastases location, since co-injection of TASCs together with colon cancer cells appeared to result in increased metastases formation in the liver, whereas in vitro preconditioning of tumor cells promoted their spreading into the lungs. Although further studies are warranted to clarify this phenomenon, it could be hypothesized that a more prolonged contact between TASCs and tumor cells is necessary to induce upregulation in the latter of specific chemokine receptors and/or adhesion molecules required for dissemination into specific secondary sites.

Following their extensive phenotypic and functional characterization, we have used colon-cancer-derived TASCs and corresponding MASCs for proteomic studies aimed at the identification of novel prognostic markers. A number of proteins were found to be significantly overexpressed in TASCs compared to MASCs. They included several markers associated to myofibroblast-like cell populations. 

Most importantly, the expression of genes encoding two members of the latter group, calponin-1 (CNN1) and tropomyosin beta chain isoform 2 (TPM2), significantly correlates with severe prognosis in colon cancer public databases and outperforms the expression of previously reported TASC-associated markers, consistent with a potentially higher clinical relevance. 

Both CNN1 and TPM2 are highly contractile proteins expressed by smooth muscle cells. Moreover, while TPM2 is also expressed in myocardial and striated muscle cells, CNN1 protein is highly expressed by pericytes, stabilizing blood vessels [33,34]. 

The expression of CNN1 gene had previously been associated with malignant transformation [35] and has recently been included, together with TPM2 gene expression, in a transcriptional regulatory network associated with colorectal cancer recurrence [36]. However, the nature of the cells expressing these genes in the tumor microenvironment was not addressed.

More interestingly, most recent studies based on single-cell gene expression analysis in colorectal cancer surgical specimens, concur in the identification of both CNN1 and TPM2 as TASC markers associated with poor prognosis [37,38]. In this respect, our findings represent an important validation, emerging from studies carried out with a totally different technological approach. Most importantly, they provide functional characterization of cells expressing these newly identified stromal markers within the colorectal cancer microenvironment, demonstrating that TASCs overexpressing CNN1 and TPM2 at protein level do directly support cancer progression by enhancing tumor cell proliferation, migration, and metastatic potential. On the other hand, the observed correspondence between data from ex vivo single-cell sequencing and our proteomic analysis of colon cancer-derived TASCs rules out the potential occurrence of significant biases upon in vitro expansion and fully supports the integrity of our experimental approach.

In addition, considering the functional specificities of CNN1 and TPM2 proteins, our data suggests that TASC biomechanical properties might be of relevance for colon cancer progression. Indeed, it has been recently shown in an in vitro model that, by exerting contractile forces, TASCs alter the organization and the physical properties of the basement membrane, thus making it permissive for cancer cell invasion [39].

Limitations of our study should be acknowledged. The number of TASC and MASC preparations used throughout our work is modest. However, their high functional relevance is strongly supported by the analysis of the orthotopic experimental model, allowing the evaluation of metastatic spread. Moreover, the analysis of large, unrelated, publicly available, databases powerfully supports the relevance of emerging findings. On the other hand, immunohistochemical validation of the prognostic significance of the identified markers is hampered by the lack of sufficiently highly specific reagents, particularly regarding TPM2. However, at least for CNN1, immunohistochemical data from recent published data [37] and a publicly available database (https://www.proteinatlas.org accessed date 14 January 2022) consistently supports the expression of target protein in colon-cancer-associated TASCs, and its prognostic significance. 

## 5. Conclusions

In conclusion, our data strongly support the notion of a key role of TASCs in colon cancer clinical course and identify TPM2 and CNN1 as novel and robust prognostic markers for colon cancer prognostication. These findings may be helpful for the identification of patients at risk of progression, possibly benefiting from additional treatments, and may pave the way towards the development of novel TASC-targeted therapeutic protocols.

## Figures and Tables

**Table 1:** Most abundant stromal-lineage-associated proteins upregulated in TASCs versus MASCs.

Protein Description	UniProt	Gene	NSAF ^1^	log2 T/M Ratio ^2^	−log10 adj *p* Value	K-M *p* Value (Median) ^3^	K-M *p* Value (Best) ^4^
Isoform 2 of Tropomyosin beta chain	P07951-2	TPM2	2.1303	1.1470	1.9303	0.0063	0.00025
Calponin-1	P51911	CNN1	0.45455	0.6931	2.0427	0.0053	0.0026
Actin, aortic smooth muscle OS	P62736	ACTA2	1.0451	0.7331	1.6676	0.14	0.0044
Transgelin	Q01995	TAGLN	2.8507	1.3696	2.0242	0.052	0.0057
Myosin regulatory light polypeptide 9	P24844	MYL9	0.29651	0.8597	2.1649	0.045	0.013
Synemin	O15061	SYNM	0.061981	0.6349	1.4197	0.098	0.021
Isoform 4 of Protein MRVI1	Q9Y6F6-4	MRVI1	0.032115	0.8740	1.7685	0.34	0.022
Cysteine and glycine-rich protein 1	P21291	CSRP1	0.62694	1.0287	1.6296	0.38	0.04
Isoform Del-1790 of Myosin light chain kinase	Q15746-6	MYLK	0.11657	0.7109	1.8684	0.47	0.052
Isoform 2 of Sorbin and SH3 domain-containing protein 2	O94875-2	SORBS2	0.034535	0.7215	1.3333	0.094	0.061
Myosin-11	P35749	MYH11	0.15314	0.7765	1.3643	0.25	0.08
Isoform 2 of PDZ and LIM domain protein 3	Q53GG5-2	PDLIM3	0.079114	1.6642	2.9757	0.1	0.1
Isoform PDE5A2 of cGMP-specific 3’,5’-cyclic phosphodie	O76074	PDE5A	0.084034	0.6554	1.4091	0.7	0.17

^1^ Normalized spectral abundance factor (NSAF). ^2^ T/M ratio = TASCs/MASCs ratio. ^3^ *p* value of log-rank test in TCGA COAD cohort (*n* = 483) dichotomized according to median value. ^4^ *p* value of log-rank test in TCGA COAD cohort (*n* = 483) dichotomized according to best cut-off.

## Data Availability

Raw mass spectrometric data used in this study and the Mascot analysis files are available via ProteomeXchange (accession code PXD005796, Username: reviewer25764@ebi.ac.uk, Password: vadeGPHV). Prognostic significance of the expression of defined genes was analyzed by using Xena (https://xenabrowser.net) website and GDC TCGA COAD database, with primary tumor samples.

## Supplementary Materials

The following supporting information can be downloaded at: https://www.mdpi.com/article/10.3390/cancers14082024/s1, Figure S1: Characterization of colon cancer-derived TASCs, Figure S2: TASCs induce upregulation of EMT-related genes in colon cancer cell lines, Figure S3: TASCs capacity to induce EMT in tumor cells depends on their differentiation state, Figure S4: Direct cell-to-cell contact between tumor cells and TASCs is required for EMT induction, Figure S5: Evaluation of tumor growth and metastasis formation upon development of orthotopic tumor xenografts, Table S1: Clinical-pathological characteristics of tumor samples from which TASCs used in experiments were derived, Table S2: List of antibodies used for flow cytometry and immunofluorescence, Table S3: Proteins significantly upregulated in TASCs versus MASCs, Table S4: Previously reported TASCs markers.
