# Supplementary material for: Identification of TPM2 and CNN1 as Novel Prognostic Markers in Functionally Characterized Human Colon Cancer-Associated Stromal Cells
Source: Cancers (Basel). 2022 Apr 16;14(8):2024. doi: 10.3390/cancers14082024 (PMC9025001; doi:10.3390/cancers14082024)
Supplement: Supplementary file 1 [file cancers-14-02024-s001.zip › cancers-1601052-supplementary.pdf]

cancers

Supplementary Material

Table S1. Clinical-pathological characteristics of tumor samples from which TASCs used in experiments were derived

Features		Frequency n (%)
**Tumor location**	Left-sided	5 (62.5)
	Right-sided	3 (37.5)
**Grade**	G1	3 (37.5)
	G2	5 (62.5)
	G3	0 (0)
	G4	0 (0)
**pT stage**	T1	1 (12.5)
	T2	1 (12.5)
	T3	6 (75)
	T4	0 (0)
**pN stage**	N0	3 (37.5)
	N1	4 (50)
	N2	1 (12.5)

Table S2. List of antibodies used for flow cytometry and immunofluorescence.

Name	Clone	Brand	Dilution used
**CD90**	5E10	BD	1:200
**CD73**	AD2	BD	1:50
**CD105**	43A3	Biolegend	1:200
**CD29**	TS2/16	Biolegend	1:200
**CD146**	SHM-57	Biolegend	1:200
**CD34**	581	BD	1:25
**CD45**	HI30	Biolegend	1:200
**CD31**	WM59	Biolegend	1:200
**CD31**	Mec 13.3	BD	1:100
**aSMA**	E184	Abcam	1:100
**aSMA**	1A4	SIGMA	1:100
**FAP**	1E5	Abcam	1:100
**FSP-1**	EPR2761(2)	Novus Biologicals	1:100
**EpCAM**	EBA-1	Biolegend, BD	1:50
**EpCAM**	9C4	Biolegend	1:200
**EpCAM**	VU1D9	Cell Signalling	1:200
**TGFβ**	9016	R&D Systems	1:50
**PDPN**	NC-08	Biolegend	1:200
**E-Cadherin**	67A4	Biolegend	1:100
**N-Cadherin**	8C11	Biolegend	1:100
**Vimentin**	D21H3	Cell Signalling	1:100
**αvβ6**	53a.2	Abcam	1:100

**Ki67**	EPR3610	Abcam	1:200

Table S3. Proteins significantly upregulated in TASCs versus MASCs.

Protein description	UniProt	Gene	NSAF[1]	log2 T/M ratio[2]	-log10 adj p–value
**Transgelin**	Q01995	TAGLN	2.8507	1.369591776	2.024216788
**Isoform 2 of Tropomyosin beta chain**	P07951-2	TPM2	2.1303	1.146955709	1.930278652
**Actin, aortic smooth muscle**	P62736	ACTA2	1.0451	0.73306496	1.667646232
**Cysteine and glycine-rich protein 1**	P21291	CSRP1	0.62694	1.028708326	1.629555599
**Isoform 1 of Vinculin**	P18206-2	VCL	0.59756	0.731613704	2.072906862
**Isoform 2 of Tropomyosin alpha-4 chain**	P67936-2	TPM4	0.50352	1.218728987	3.660414337
**Calponin-1**	P51911	CNN1	0.45455	0.693143643	2.042665302
**Four and a half LIM domains protein 2**	Q14192	FHL2	0.45161	0.61098913	2.11323767
**Myosin regulatory light polypeptide 9**	P24844	MYL9	0.29651	0.859685477	2.164914646
**Superoxide dismutase [Mn], mitochondrial**	P04179	SOD2	0.24324	1.430159513	1.375014938
**Myosin-11**	P35749	MYH11	0.15314	0.776470331	1.364268629
**Aldehyde dehydrogenase X, mitochondrial**	P30837	ALDH1B1	0.14313	0.665703034	1.894322199
**Isoform 8 of Tropomyosin alpha-1 chain**	P09493-8	TPM1	0.12676	0.659642581	1.300274496
**Isoform Del-1790 of Myosin light chain kinase**	Q15746-6	MYLK	0.11657	0.710862859	1.868401708
**Isoform PDE5A2 of cGMP-specific 3',5'-cyclic phosphodiesterase**	O76074	PDE5A	0.084034	0.655432099	1.409133562
**Tubulin beta-2A chain**	Q13885	TUBB2A	0.083146	0.622955915	1.885446624
**Isoform 2 of PDZ and LIM domain protein 3**	Q53GG5-2	PDLIM3	0.079114	1.664154334	2.97566031
**Intercellular adhesion molecule 1**	P05362	ICAM1	0.073308	1.928758837	2.40873185
**von Willebrand factor A domain-containing protein 5A**	O00534	VWA5A	0.062341	0.608349475	2.166147035
**Synemin**	O15061	SYNM	0.061981	0.634940911	1.419658575
**Cell surface glycoprotein MUC18**	P43121	MCAM	0.055728	1.088975189	3.069731623
**Isoform 2 of CD82 antigen**	P27701	CD82	0.053719	0.905728254	3.03133314
**Isoform 2 of Sarcolemmal membrane-associated protein**	Q14BN4-2	SLMAP	0.039241	1.408815068	1.870052561
**Peptidyl-prolyl cis-trans isomerase F, mitochondrial**	P30405	PPIF	0.038647	0.598108968	1.346828397
**Profilin-2**	P35080	PFN2	0.035714	0.609263793	2.21497501
**Isoform 2 of Sorbin and SH3 domain-containing protein 2**	O94875-2	SORBS2	0.034535	0.721487744	1.333348543
**TBC1 domain family member 2A**	Q9BYX2	TBC1D2	0.032328	0.676395709	2.311535917
**Isoform 4 of Protein MRVI1**	Q9Y6F6-4	MRVI1	0.032115	0.873990944	1.768513891
**Interferon-induced GTP-binding protein Mx1**	P20591	MX1	0.030211	1.0648971	1.657913505
**Annexin A3**	P12429	ANXA3	0.027864	1.445390134	1.433163419
**Interferon-induced protein with tetratricopeptide repeats 3**	O14879	IFIT3	0.026531	1.102235479	2.296449251
**Na(+)/H(+) exchange regulatory cofactor NHE-RF1**	O14745	SLC9A3R1	0.02514	0.613075965	1.37828742
**Integrin alpha-3**	P26006	ITGA3	0.024738	1.157701255	2.045437517
**GDP-mannose 4,6 dehydratase**	O60547	GMDS	0.024194	0.642798867	2.121599529
**Pyroglutamyl-peptidase 1**	Q9NXJ5	PGPEP1	0.023923	0.670435865	1.539127543
**Isoform 2 of Redox-regulatory protein FAM213A**	Q9BRX8	FAM213A	0.022936	0.97053097	2.749372394
**5'-nucleotidase domain-containing protein 3**	Q86UY8	NT5DC3	0.021898	1.023362305	1.526041891
**Isoform 2 of EGF-like repeat and discoidin I-like domain-containing protein 3**	O43854-2	EDIL3	0.019149	0.89064351	1.892695166
**C-X-C motif chemokine 14**	O95715	CXCL14	0.018018	0.634647798	1.388565252
**Junctional protein associated with coronary artery disease**	Q9P266	KIAA1462	0.014717	0.815090113	2.01049799
**Isoform 2 of Cordon-bleu protein-like 1**	Q53SF7	COBLL1	0.01458	1.164800305	1.844518957
**Beta-2-glycoprotein 1**	P02749	APOH	0.014493	0.6092497	1.77113088
**Squalene synthase**	P37268	FDFT1	0.014388	0.58771711	1.544439462
**Isoform 3 of Endoplasmic reticulum aminopeptidase 2**	Q6P179-3	ERAP2	0.014208	0.805458491	1.351017808
**Eukaryotic translation initiation factor 1A, Y-chromosomal**	O14602	EIF1AY	0.013889	2.116864833	1.704081173
**Isoform 2 of Mitogen-activated protein kinase kinase kinase MLT**	Q9NYL2-2	ZAK	0.013187	1.284414688	1.462184202
**Uncharacterized protein C15orf52**	Q6ZUT6	C15orf52	0.013109	0.59257026	1.329147307
**Fructosamine-3-kinase**	Q9H479	FN3K	0.012945	0.599023216	1.754148201
**Isoform 2 of Receptor expression-enhancing protein 2**	Q9BRK0-2	REEP2	0.011811	0.786168972	1.604696573
**Desmoglein-2**	Q14126	DSG2	0.010733	1.113110737	1.413378891
**snRNA-activating protein complex subunit 5**	O75971	SNAPC5	0.010204	0.660994283	2.095003198
**NADP-dependent malic enzyme, mitochondrial**	Q16798	ME3	0.0099338	0.650368603	1.508888857

**Isoform 2 of Dual specificity protein phosphatase 22**	Q9NRW4-2	DUSP22	0.0097561	0.588674525	1.915816667
**Ras-related protein Rab-27B**	O00194	RAB27B	0.0091743	0.73147857	1.318090928
**Synaptopodin-2**	Q9UMS6	SYNPO2	0.0091491	1.152318809	2.324063334
**Isoform 2 of Neuronal growth regulator 1**	Q7Z3B1-2	NEGR1	0.0088496	0.623248463	1.764740807
**GMP reductase 1**	P36959	GMPR	0.0086957	0.666657304	1.367637382
**Ephrin-B1**	P98172	EFNB1	0.0086705	0.681586149	2.407998395
**Neural cell adhesion molecule 2**	O15394	NCAM2	0.0083632	1.075789734	2.521357996
**Complement C1q tumor necrosis factor-related protein 5**	Q9BXJ0	C1QTNF5	0.0082305	0.63021796	1.901545219
**N-acetylgalactosaminyltransferase 7**	Q86SF2	GALNT7	0.0076103	0.765409681	1.382277499
**Isoform I of Septin-6**	Q14141-4	SEPT6	0.0070258	1.174872486	2.666389117
**Collectin-12**	Q5KU26	COLEC12	0.0067385	0.839409378	2.703170057
**Isoform 3 of Alpha-actinin-1**	P12814-3	ACTN1	0.0067265	0.653256818	1.551684606
**Ninjurin-1**	Q92982	NINJ1	0.0065789	0.661403942	2.378112927
**Actin-binding LIM protein 1**	O14639	ABLIM1	0.0064267	0.927887461	1.874323618
**Sodium-dependent phosphate transporter 2**	Q08357	SLC20A2	0.006135	1.181213583	1.390201729
**Pro-cathepsin H**	P09668	CTSH	0.0059701	0.960753372	2.141276676
**Isoform 2 of Growth arrest-specific protein 6**	Q14393-2	GAS6	0.0058997	1.118955025	2.436266589
**Isoform 2 of Serine protease 23**	O95084-2	PRSS23	0.005698	1.065393153	3.139927518
**Maestro heat-like repeat-containing protein family member 1**	Q8NDA8	MROH1	0.0054845	0.605337925	1.785431841
**Isoform 2 of Solute carrier family 2, facilitated glucose transporter member 6 GN=SLC2A6**	Q9UGQ3	SLC2A6	0.0044944	0.725661842	1.563477258
**Isoform 2 of DENN domain-containing protein 3**	A2RUS2	DENND3	0.004363	1.50656138	2.896299523
**Junctophilin-2**	Q9BR39	JPH2	0.0043103	1.917760552	2.367822915
**Isoform 2 of Multiple PDZ domain protein**	O75970-2	MPDZ	0.0039196	0.653794964	1.713013374
**Phosphoinositide-3-kinase-interacting protein 1**	Q96FE7	PIK3IP1	0.0038023	1.004159992	2.725592963
**Isoform 2 of Disheveled-associated activator of morphogenesis 2**	Q86T65	DAAM2	0.0037488	0.625118135	1.526883815
**Protein FAM84A**	Q96KN4	FAM84A	0.0034247	1.713092913	3.016708531
**Kelch-like protein 21**	Q9UJP4	KLHL21	0.0033501	1.058100137	2.145910136
**Isoform 2 of Xenotropic and polytropic retrovirus receptor 1**	Q9UBH6	XPR1	0.0031696	0.690162944	2.434796638
**Isoform 3 of Bcl-2-like protein 12**	Q9HB09-3	BCL2L12	0.003003	0.628582242	1.521943617
**Isoform LCRMP-1 of Dihydropyrimidinase-related protein 1**	Q14194-2	CRMP1	0.0029155	0.668038969	1.959832867
**Beta-1,4-galactosyltransferase 4**	O60513	B4GALT4	0.002907	0.805925093	1.660352963
**Zinc finger BED domain-containing protein 1**	O96006	ZBED1	0.0028818	0.835505836	1.430145763
**Isoform 5 of Disabled homolog 2-interacting protein**	Q5VWQ8	DAB2IP	0.0026502	0.613816689	2.113292909
**Isoform 2 of Solute carrier family 2, facilitated glucose transporter member 4**	P14672	SLC2A4	0.0024096	1.29691691	3.180367016
**Phosphatidylcholine:ceramide cholinephosphotransferase 1**	Q86VZ5	SGMS1	0.0023866	0.771686724	1.828680742
**Isoform 5 of CCR4-NOT transcription complex subunit 4**	O95628-5	CNOT4	0.0023095	0.651292955	2.118262217
**Integral membrane protein GPR155**	Q7Z3F1	GPR155	0.0022989	0.640027315	2.147542393
**Interferon-induced protein 44**	Q8TCB0	IFI44	0.0022523	0.749256786	1.885455005
**Disintegrin and metalloproteinase domain-containing protein 19**	Q9H013	ADAM19	0.0020942	0.594223888	1.549447658
**Ribosomal protein S6 kinase beta-2**	Q9UBS0	RPS6KB2	0.0020747	0.64001956	1.501764907
**Isoform 4 of Rho guanine nucleotide exchange factor 10-like protein**	Q9HCE6-4	ARHGEF10L	0.0020367	0.901245663	2.134385685
**Amphoterin-induced protein 2**	Q86SJ2	AMIGO2	0.0019157	0.585036598	2.038956885
**SH3KBP1-binding protein 1**	Q8TBC3	SHKBP1	0.0014144	0.646003345	1.615812668
**Isoform 2 of Telomere-associated protein RIF1**	Q5UIP0-2	RIF1	0.0012265	2.074826105	4.306720488
**Isoform 2 of Neural cell adhesion molecule 1**	P13591-1	NCAM1	0.0011792	0.725111612	1.371141475
**DNA (cytosine-5)-methyltransferase 3A**	Q9Y6K1	DNMT3A	0.0010965	0.64407483	1.914928422
**Isoform 2 of Nesprin-2**	Q8WXH0	SYNE2	0.0010135	0.960456358	1.342666208
**Sterol regulatory element-binding protein 2**	Q12772	SREBF2	0.00087642	0.676395721	1.419569465
**Phosphatidylinositol 4-phosphate 3-kinase C2 domain-containing subunit beta**	O00750	PIK3C2B	0.000612	0.650472734	2.377016406
**Isoform 2 of Dedicator of cytokinesis protein 4**	Q8N1I0	DOCK4	0.00051626	0.833251423	1.319558447
**Isoform 2 of Myosin-14**	Q7Z406-6	MYH14	0.00049116	0.635395693	1.820246093
**Isoform 1 of Striated muscle preferentially expressed protein kinase**	Q15772	SPEG	0.00031027	0.828858327	1.748434026

[1] Normalized Spectral Abundance Factor (NSAF)
[2] T/M ratio=TASCs/MASCs ratio

Table S4. Previously reported TASC markers

Protein description	UniProt	Gene	NSAF[1]	log2 T/M ratio	-log10 adj p–value	K-M p value (median)[2]	K-M p value (best)[3]
Protein S100-A4 FSP1	P26447	FSP1 S100A4	0.24752	0.01300	0.0057	0.33	0.099
Insulin-like growth factor-binding protein 7	Q16270	IGFBP7	0.19858	0.18416	0.2418	0.45	0.12
Isoform 2 of Procollagen-lysine,2-oxoglutarate 5-dioxygenase 2	O00469-2	PLOD2	0.11478	0.20681	0.0684	0.77	0.12
Prolyl endopeptidase FAP	Q12884	FAP	0.048684	0.16887	0.2649	0.41	**0.043**
Isoform 4 of Caldesmon	Q05682-4	CALD1	0.033457	-0.05515	0.3190	0.49	**0.006**
Periostin	Q15063	POSTN	0.01555	0.33831	0.0894	0.092	**0.017**
Isoform 4 of Podoplanin	Q86YL7-4	PDPN	0.012712	-0.32283	0.1487	0.71	0.25
Lysyl oxidase homolog 2	Q9Y4K0	LOXL2	00.011628	0.11567	0.6778	0.21	0.14

[1] Normalized spectral abundance factor (NSAF).[2] T/M ratio = TASCs/MASCs ratio.[3] *p* value of log-rank test in TCGA COAD cohort (*n* = 483) dichotomized according to median value. [4] *p* value of log-rank test in TCGA COAD cohort (*n* = 483) dichotomized according to best cut-off.

(a)

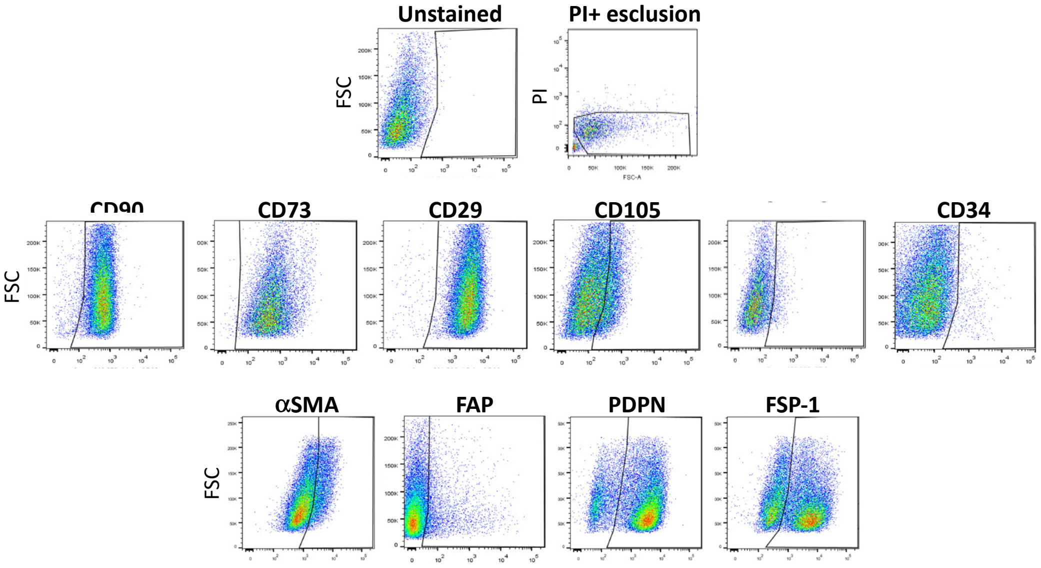

(b)

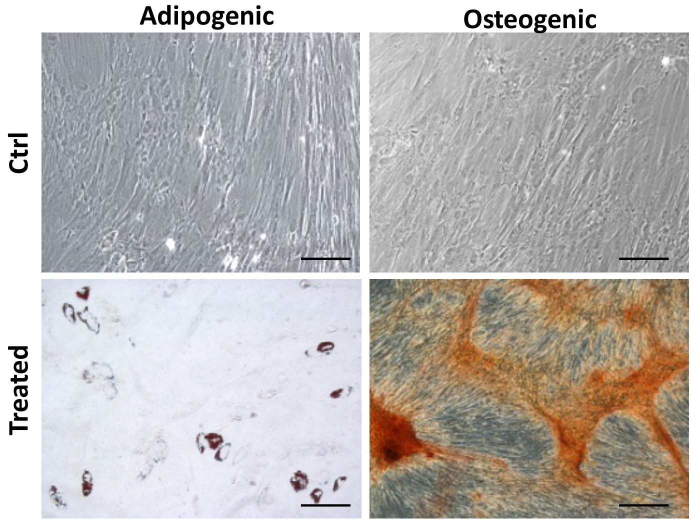

Figure S1. Characterization of colon cancer-derived TASCs. (**a**) Representative phenotypical analysis of TASCs by flow cytometry. Gates are defined according to the corresponding unstained sample. Dead cells are excluded based on PI incorporation. (**b**) Representative pictures of TASC differentiation into adipogenic and osteogenic lineages, as evaluated by Oil Red O and Alizarin Red staining, respectively. Magnification 20x, scale bar 100μm. Data refer to 1 representative experiment out of 4 independently performed with 4 different TASC samples, with similar results.

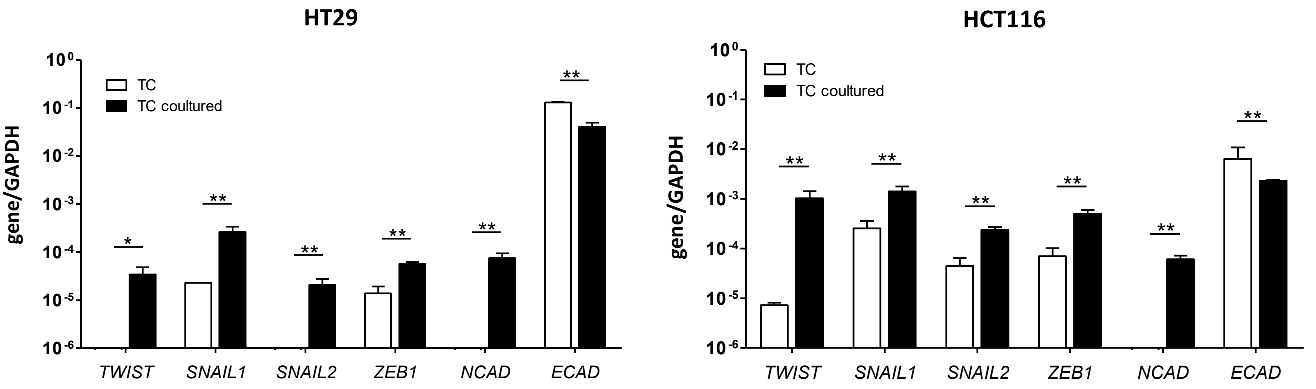

Figure S2. TASCs induce upregulation of EMT-related genes in colon cancer cell lines. Quantitative RT-PCR analysis of EMT-related genes in colon cancer cells from the indicated cell lines cultured alone (TC) or cocultured with TASCs (TC cocultured). Mean ± SD from triplicates of cultures are shown. *p≤0.05; **p≤0.01.

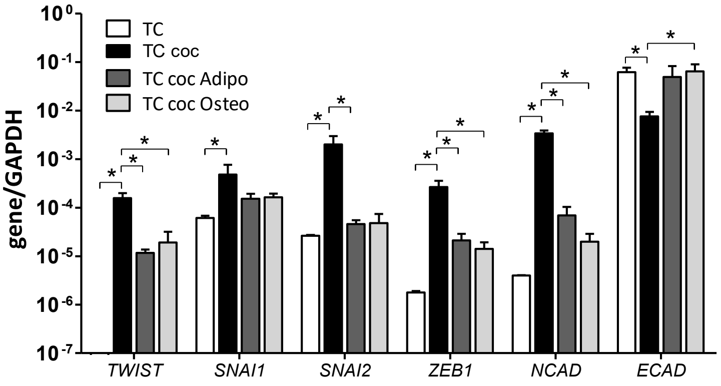

Figure S3. TASCs capacity to induce EMT in tumor cells depends on their differentiation state. Quantitative RT-PCR analysis of the expression of EMT-related genes in LS180 cells cultured alone (TC), with TASCs (TC coc), or with TASCs pre-differentiated into adipocytes (TC coc Adipo) or osteocytes (TC coc Osteo). Cumulative data (mean ± SD) from four cultures performed with two different TASC samples are shown. Statistical significance of results was evaluated by Mann-Whitney test (*$p \leq 0.05$ **$p \leq 0.01$).

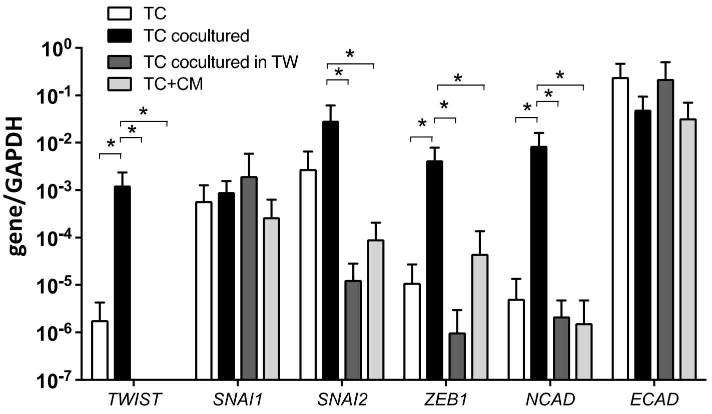

Figure S4. Direct cell-to-cell contact between tumor cells and TASCs is required for EMT induction. Quantitative RT-PCR analysis of the expression of EMT-related genes in LS180 cells cultured alone (TC), with TASCs (TC cocultured), with TASCs in transwell plates (TC cocultured in TW), or alone in the presence of TASC-conditioned medium (CM). Data (mean ± SD) refer to three independent experiments, performed with TASCs isolated from three different CRC samples. Statistical significance was evaluated by Mann-Whitney test (*$p < 0.05$).

(a)

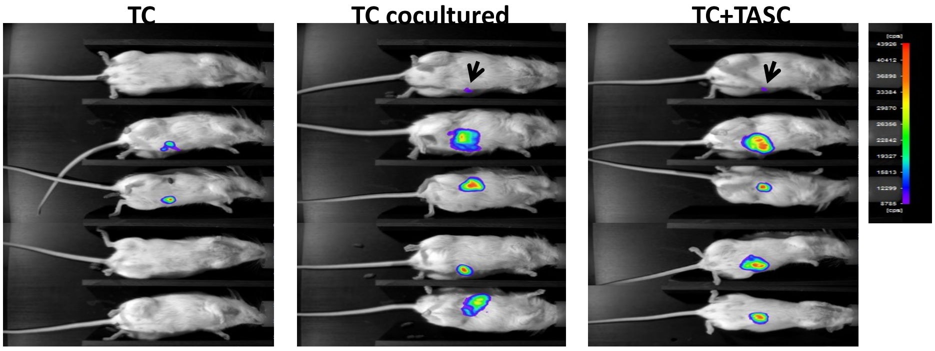

(b)

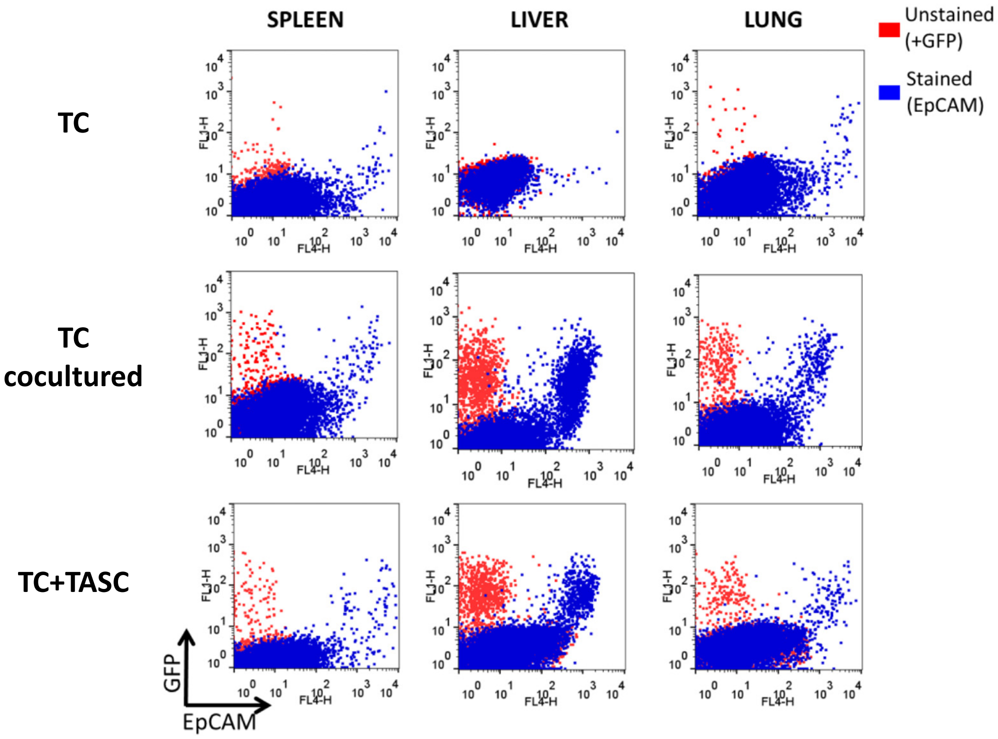

Figure S5. Evaluation of tumor growth and metastasis formation upon development of orthotopic tumor xenografts. (**a**) Representative images of tumor detection by measurement of luciferase activity in NSG mice upon orthotopic i.c. injection of GFP/Luc-LS180 cells alone (TC), cocultured with TASCs and sorted prior to injection (TC cocultured), or cultured alone and admixed with TASCs prior to injection (TC+TASC). Images are displayed as pseudo-color images of peak bioluminescence, with variations in color representing light intensity at a given location. The color bars indicate relative signal intensity. (**b**) Representative analysis by flow cytometry of cell suspensions from spleens, lungs and livers of tumor bearing mice injected as detailed above. Metastatic tumor cells are detected as GFP+ in unstained samples (red) or as GFP+ EpCAM+ cells upon staining with EpCAM-specific antibodies (blue).
